# Annexin A2 Expression in the Aerogenous Spread of Pulmonary Invasive Mucinous Adenocarcinoma with Gastric Lineage

**DOI:** 10.1155/2020/2492636

**Published:** 2020-05-18

**Authors:** Kazumori Arai, Tomohiro Iwasaki, Chinatsu Tsuchiya, Akihiro Sonoda

**Affiliations:** ^1^Department of Pathology, Shizuoka General Hospital, 4-27-1 Kitaando, Aoi-ku, Shizuoka 420-0881, Japan; ^2^Department of Clinical Research, Shizuoka General Hospital, Japan

## Abstract

Spread through air spaces (STAS) is a unique form of lung cancer progression associated with a worse prognosis. However, the mechanisms underlying STAS and the associated proteins remain unclear. Annexin A2 (ANX A2), which is a membrane-binding protein involved in cell adhesion, is known to promote cancer invasion. In this report, we describe the immunohistochemical analysis of ANX A2 expression in an invasive mucinous adenocarcinoma (IMAC) resected from a 63-year-old man in whom the tumor cells had detached from the alveolar wall and exhibited STAS. At the detachment site, we observed cytoplasmic ANX A2 positivity on the basal side and in the exfoliative gap, as well as reduced collagen IV positivity expression. This biomarker pattern suggested an IMAC with gastric lineage. We hypothesize that ANX A2 is secreted from the basal sides of tumor cells and induces tumor cell detachment by degrading the basement membrane. A further comparison of this case with an IMAC with nongastric lineage suggested the following probabilities: (1) ANX A2 likely contributes to STAS in a manner that is dependent on its subcellular localization. (2) Both the subcellular localization of ANX A2 and the detachment site depend on tumor cell characteristics, including the biomarker immunophenotype.

## 1. Introduction

Spread through air spaces (STAS) is a unique form of lung cancer progression from the primary tumor site via the airways [1, 2]. This new category is included in the definition of lung adenocarcinoma (AC) invasion, along with stromal (including vascular) and pleural invasion [1]. STAS is also considered the origin of aerogenous metastasis (AM) [1, 2]. Although STAS has elicited great interest in the field of lung cancer [3–7], its status as either a true invasive form or a technical artifact of sample processing remains controversial [3]. However, recent reports have provided evidence that favors STAS as a true invasive pattern and have demonstrated a significant association with a worse prognosis [4–7]. Furthermore, STAS is associated with a higher risk of locoregional recurrence, thus preventing limited resection [4–6]. The inhibition of STAS is an important research issue, but the underlying pathogenic mechanisms remain unclear. We believe that the ability to inhibit STAS will depend on an understanding of the protein(s) involved in the processes of tumor cell detachment into alveolar spaces and adhesion to the alveolar wall.

Invasive mucinous AC (IMAC) frequently progresses via STAS/AM [8]. Recently, we reported the expression of annexin A2 (ANX A2), which is a Ca^2+^/membrane-binding protein in a case of IMAC with STAS/AM [9]. To the best of our knowledge, our report was the first to indicate the involvement of this protein in STAS/AM. In the reported case, tumor cell detachment occurred at the tip of a villous-shaped tumor component that had projected into the alveolar spaces [9]. In contrast to our previous report, other researchers described tumor cell detachment from the basement membrane (BM) of the alveolar wall as a common origin of STAS/AM from IMAC [10, 11].

ANX A2 is expressed in various cells and is involved in cell adhesion, cell proliferation, and cell polarity within the cytoplasm and plasma membranes [12, 13]. This protein is also expressed in various cancers, including lung cancer, and extracellular membrane-bound ANX A2 acts as a plasminogen activator to promote angiogenesis and cancer cell invasion in the stroma [14, 15]. The other functions of ANX A2 in cancer are currently under investigation [15]. We hypothesized that ANX A2 may also be involved in the STAS/AM of lung AC and that the observed discrepancies in IMAC detachment sites would result from differences in ANX A2 expression or subcellular localization. In the current report, we immunohistochemically examined the expression of ANX A2 in a case of IMAC in which tumor cell detachment occurred from the alveolar wall, consistent with previous reports [10, 11].

## 2. Case Presentation

**Figure 1:**
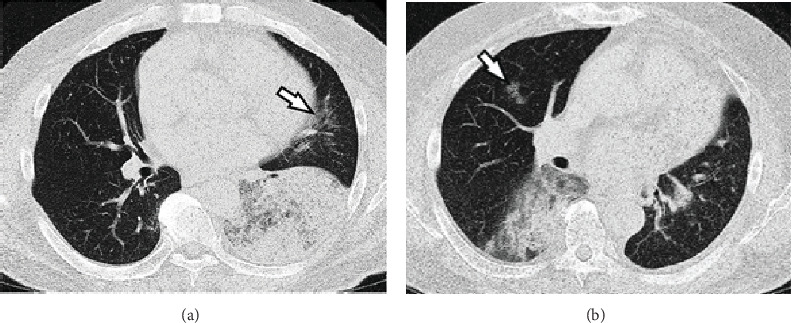
Axial CT images. (a) A chest CT image obtained 7 days after the initial visit shows a large infiltrative shadow, as well as a small infiltrative shadow that is suspected to be a skip lesion (arrow). (b) A chest CT image obtained 2 months after left lobectomy shows an infiltrative shadow with a skip lesion (arrow) in the right lung.

The subject of this case study provided written informed consent for the publication of the case details. A 63-year-old man with no history of smoking presented with fever, cough, and mucinous sputum. Chest computed tomography (CT) revealed a large infiltrative shadow that is approximately 10 cm in size in the lower lobe of the left lung. No enlargement of the hilar or mediastinal lymph nodes was observed. The differential diagnosis was challenging, and pneumonia or IMAC was suspected [16]. Seven days later, chest CT demonstrated the expansion of the shadow and a small infiltrative shadow suggestive of a skip lesion (Figure 1(a)). The patient was then hospitalized immediately. Full-body CT did not reveal any metastatic lesions in the right lung or extrapulmonary organs. Considering that a transbronchial lung biopsy revealed a mucus-producing AC, the patient underwent thoracoscopic lobectomy with no adjuvant therapy.

Two months postoperatively, a follow-up chest CT revealed an infiltrative shadow with an approximate diameter of 6 cm and a skip lesion in the lower lobe of the right lung (Figure 1(b)). A right lung biopsy revealed an AC that shared cell morphologic features with the left lung tumor. The comprehensive clinical course led to a clinical suspicion that the right lung tumor was an AM from the left lung tumor [17]. No metastatic lesion was detected in the left lung. The patient did not undergo surgery for the right lung tumor and instead received various chemotherapy regimens for 30 months, including 11 cycles of paclitaxel, carboplatin, and bevacizumab; 10 cycles of pemetrexed; and 16 cycles of docetaxel. However, no distinct therapeutic effect was observed, and CT revealed a further enlargement of the shadow. The patient developed right pleuritis carcinomatosa at 31 months after the start of chemotherapy and died of respiratory failure 36 months after surgery. An autopsy was not performed.

In addition to a routine pathological analysis, tissue from the left-sided tumor was subjected to immunohistochemical analyses to detect the expression of various proteins, including ANX A2 and collagen IV. Immunohistochemistry was performed using Leica Bond-Max (Leica Biosystems, Australia) and a previously described ANX A2 antibody and staining protocol [9]. A mouse antihuman collagen IV antibody (clone CIV 22, DAKO, USA) was used as the primary antibody at a dilution ratio of 1 : 25 after a 5 min treatment with proteinase K. Only routine immunohistochemistry was performed on the right lung tumor because of the small amount of available tissue.

**Figure 2:**
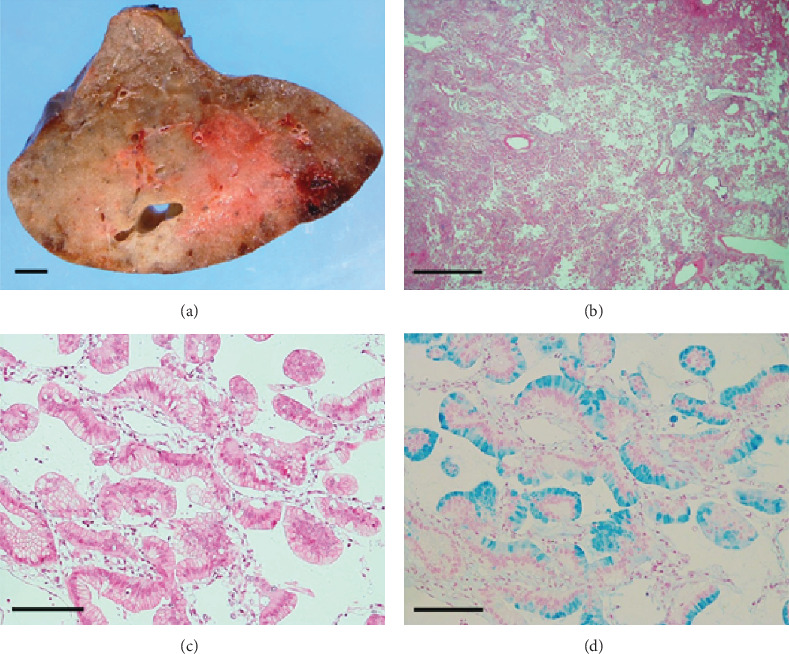
Left lung cancer I. (a) Gross view showing an ill-defined tumor with a diffuse pneumonia-like consolidation. Scale bar: 1 cm. (b) The tumor cells exhibit lepidic or papillary growth, with detached tumor cells scattered in the alveolar spaces. Hematoxylin and eosin (H&E) staining, scale bar: 2 mm. (c) Columnar tumor cells with intracytoplasmic vacuoles are shown in a monolayer arrangement. H&E stain, scale bar: 200 *μ*m. (d) Multiple mucin-producing tumor cells are visible. Alcian blue stain, scale bar: 200 *μ*m.

**Figure 3:**
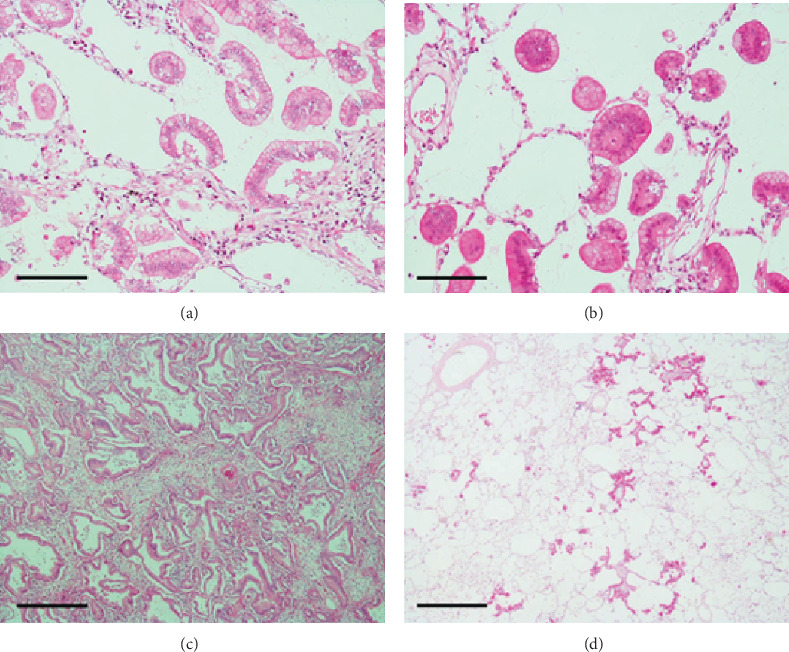
Left lung cancer II. (a) Small groups of tumor cells that detached from the alveolar wall. Only a slight inflammatory cell infiltration is visible. H&E stain, scale bar: 200 *μ*m. (b) Detached tumor cells with a micropapillary pattern float within the alveolar spaces. H&E stain, scale bar: 200 *μ*m. (c) The invasive component exhibits glandular or papillary growth. H&E stain, scale bar: 1 mm. (d) Many scattered tumor cell aggregates that detached from the primary tumor are visible within the air spaces and on the alveolar wall. The latter aggregates exhibit lepidic or papillary growth that is suggestive of microscopic aerogenous metastases. H&E stain, scale bar: 2 mm.

Macroscopically, the resected tumor was ill-defined and gelatinous with a diffuse pneumonia-like consolidation (Figure 2(a)). Histopathologically, the majority of the tumor cells were columnar, contained varying amounts of intracytoplasmic mucins, and exhibited lepidic or papillary growth in a single-layer arrangement (Figures 2(b)–2(d)). Small groups of tumor cells had detached from the alveolar wall and were scattered diffusely (Figure 3(a)). Tumor cell aggregates floating within the alveolar spaces were round-shaped but had apical–basal polarity (Figure 3(b)). The apical surface of the tumor cells was located at the outer edge of the aggregates but naturally lacked central fibrovascular cores (Figure 3(b)), corresponding to the micropapillary pattern of the three morphological patterns of STAS [6].

Slight neutrophil and macrophage infiltration was observed at the detachment site. No desmoplastic reaction was observed.

Invasive foci with a maximum diameter of 2.2 cm were observed in the tumor. These foci had a glandular or papillary structure (Figure 3(c)) and greatly reduced levels of intracytoplasmic mucins. However, the nonmucinous component accounted for <10% of the tumor. Lymphatic and vascular permeation and pleural invasion were not observed. Many scattered tumor cell aggregates that had detached from the main tumor were observed within the air spaces and on the alveolar wall. The latter aggregates exhibited lepidic or papillary growth that is suggestive of microscopic AMs (Figure 3(d)) [2]. Tumor cell detachment was also often observed in the larger foci.

**Figure 4:**
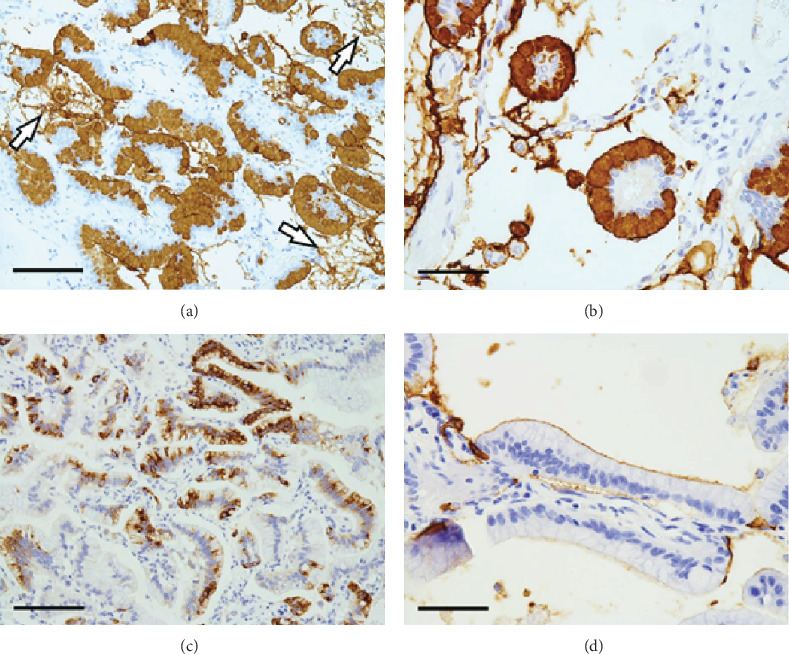
Immunostaining of mucin glycoproteins in the left lung tumor. (a) Diffuse cytoplasmic positivity is visible in the lepidic or papillary component, and positive staining is present in the alveolar spaces (arrows). MUC 5AC immunostain, scale bar: 200 *μ*m. (b) Floating tumor cells exhibit the same positive reaction. MUC 5AC immunostain, scale bar: 100 *μ*m. (c) Some tumor cells exhibit cytoplasmic positivity. MUC 6 immunostain, scale bar: 200 *μ*m. (d) In some cells, positive staining is visible on the cell surfaces but not in the cytoplasm. MUC 1 immunostain, scale bar: 100 *μ*m.

In the left lung tumor, the majority of tumor cells were positive for MUC 5AC (Figures 4(a) and 4(b)) and CK7 (data not shown). Abundant positive MUC 5AC staining was also observed in the alveolar spaces (Figures 4(a) and 4(b)). Some tumor cells exhibited intracytoplasmic positivity for MUC 6 (antibody clone CLH 5, Abnova, Taiwan; Figure 4(c)). Additionally, some tumor cells exhibited a positive MUC 1 reaction at the cell surface but not in the cytoplasm (Figure 4(d)), in contrast to our previously reported case [9]. The tumor cells were negative for MUC 2, CK20, TTF-1, Napsin A, CDX2, and ALK (data not shown). The clone and manufacturer of each antibody, except MUC6, have been described elsewhere [9]. The immunohistochemical results of the right lung tumor were similar to those of the left lung tumor.

**Figure 5:**
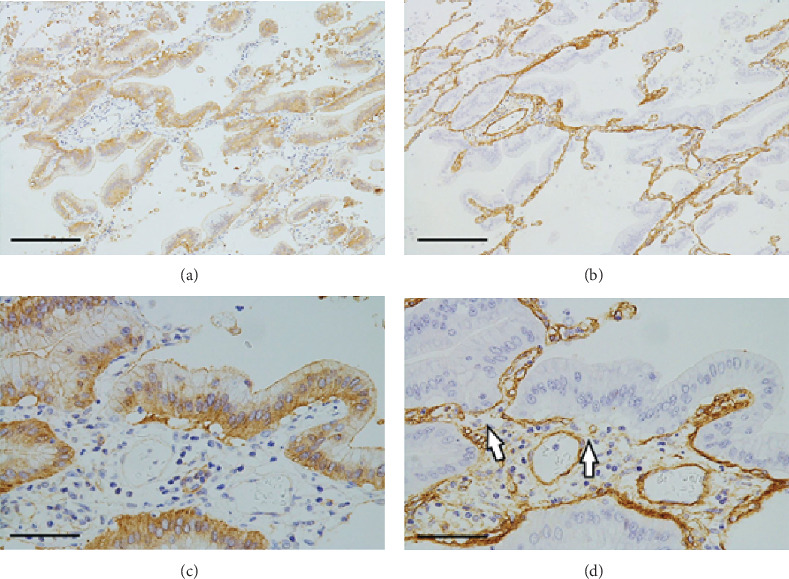
ANX A2 and collagen IV immunostaining of the lepidic or papillary component of the left lung tumor. (a) Tumor cells exhibit cytoplasmic ANX A2 positivity on the basal side. ANX A2 immunostain, scale bar: 400 *μ*m. (b) A linear positive collagen IV reaction corresponding to the basement membrane is seen on the surfaces of both alveolar and vessel walls. Collagen IV immunostain, scale bar: 400 *μ*m. (c) Detached tumor cells also exhibit cytoplasmic ANX A2 positivity on the basal side but not at the cell–cell borders. Positive staining is also observed in the exfoliative gap. ANX A2 immunostain, scale bar: 100 *μ*m. (d) Weak or no collagen IV positivity is visible at the detachment site (arrows). Collagen IV immunostain, scale bar: 100 *μ*m.

**Figure 6:**
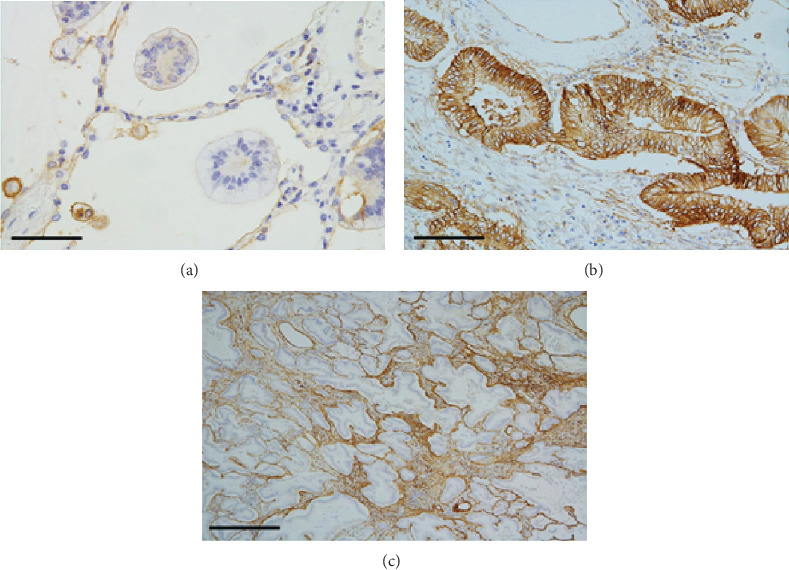
Floating tumor cells (a) and invasive components (b and c) of the left lung tumor. (a) No distinct ANX A2 positivity is visible in the floating tumor cells within alveolar spaces. ANX A2 immunostain, scale bar: 100 *μ*m. (b) Stronger cytoplasmic ANX A2 positivity is observed on the basal side, and positive staining is visible at the cell–cell borders. ANX A2 immunostain, scale bar: 200 *μ*m. (c) The desmoplastic stroma exhibits positive collagen IV staining, particularly around the tumor cells. Collagen IV immunostain, scale bar: 1 mm.

As shown in Figures 5 and 6 (5(a), 5(c), 6(a), and 6(b) show ANX A2 staining), granular cytoplasmic ANX A2 positivity was observed on the basal sides of tumor cells exhibiting lepidic or papillary growth (Figure 5(a)), whereas no positive signal was detected at the cell–cell borders. Some tumor cells also exhibited weak ANX A2 positivity near the cell surfaces. A similar expression pattern was observed in the detached tumor cells, and positive expression was also observed in the exfoliation gap (Figure 5(c)). Noticeably, weaker ANX A2 positivity was observed in the tumor cells floating within the alveolar spaces (Figure 6(a)). In the invasive component, stronger cytoplasmic positivity on the basal side was observed relative to that observed in the lepidic or papillary growth component (Figure 6(b)), and membranous positivity was observed on the cell–cell borders (Figure 6(b)).

As shown in Figures 5 and 6 (5(b), 5(d), and 6(c) show collagen IV staining), the lepidic or papillary area exhibited linear positive collagen IV staining corresponding to the BM, which was detected in both the surfaces of the alveolar and vessel walls (Figure 5(b)). However, either weak positivity or no signal was observed at the detachment site (Figure 5(d)). In the invasive area, the desmoplastic stroma exhibited positive staining for collagen IV (Figure 6(c)), thus suggesting the presence of cancer-associated fibroblasts and neogenic collagen [18, 19]. No positive reactions were observed in the tumor cells irrespective of invasiveness.

**Figure 7:**
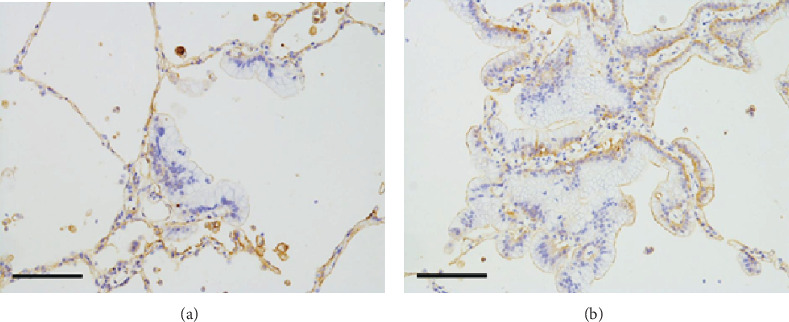
ANX A2 immunostaining of microscopic aerogenous metastases in the left lung. (a) No distinct positivity is visible in the minute foci. Scale bar: 200 *μ*m. (b) Basal-side cytoplasmic positivity is detected in the larger metastatic foci. Scale bar: 200 *μ*m.

In an analysis of the AM, no positive reaction to ANX A2 beyond weak staining near the cell surface was observed in the minute foci (i.e., tumor cells that had newly adhered to the alveolar wall; Figure 7(a)). However, the staining patterns in larger foci became similar to those observed in the lepidic or papillary growth component of the primary lesion (Figure 7(b)). Similar findings were observed at the detachment site. Regarding collagen IV, the same findings as those of lepidic or papillary growth component of primary lesion were seen. Similar findings were also observed at the detachment site (data not shown).

## 3. Discussion

ANX A2 expression in the STAS/AM of the present tumor differed slightly from that in our previously reported case [9], particularly with regard to the following four points. (1) At the tumor cell detachment site, ANX A2 positivity was observed in the cytoplasm on the basal side and in the exfoliative gap. (2) No positive staining of the cell–cell borders was observed in any component, except the invasive component. (3) No distinct ANX A2 positivity was observed in either the tumor cell aggregates floating within alveolar spaces or the minute foci of AM. (4) No ANX A2 positivity was observed in the intracytoplasmic mucus. These discrepancies are likely attributable to differences in the cellular characteristics and growth patterns between the tumors. In the present case, both the mucin immunophenotype and negative immunostaining for TTF-1 and CDX 2 suggest an IMAC with gastric lineage [20–22]. The IMAC with gastric lineage was first reported as a mucinous bronchioloalveolar carcinoma, with cell morphology mimicking the mucous cells of gastric pyloric mucosa and with its mucin immunophenotype [21, 22]. Recently, Sonzogni et al. have classified the lineage of IMACs based on their immunophenotype, histology, and genetic alterations [20]. The IMAC with gastric lineage is characterized by expressions of both membranous MUC 1 and cytoplasmic MUC 6 and is suggested to be derived from the distal terminal bronchiole [20]. In contrast, our previously reported case was negative for subsequent MUC 6 immunostaining (data not shown) and showed nontypical immunohistochemical results for any of the classification by Sonzogni et al. [9, 20]. If the previous case is categorized, it is considered to exhibit characteristics of the pancreatobiliary type in terms of the classification of the intraductal papillary mucinous neoplasms of the pancreas, particularly given the intracytoplasmic expressions of both MUC 1 and MUC 5AC in the AC [9, 23].

ANX A2 moves inside the cell according to its roles [13]. Cytoplasmic ANX A2 is involved in cytoskeleton remodeling, exocytosis, and endocytosis [13]. In addition, a part of ANX A2 binds to the extracellular surface-membrane or is secreted [13]. This protein is expressed aberrantly in various cancers [14, 15] and may also be secreted [14]. A recent report further suggested that secreted ANX A2 contributes to the proteolysis of the extracellular matrix [24]. In the present tumor, we observed reduced collagen IV staining at the detachment site, thus leading us to consider that the ANX A2 secreted from the basal sides of tumor cells might induce cell detachment by degrading the BM [13–15, 24]. Therefore, in IMAC, the detachment site of the tumor cells and the subcellular localization of ANX A2 might differ depending on the immunophenotype of the detected biomarkers [9, 20].

Indistinct membranous ANX A2 positivity in contact with the alveolar wall suggests poor capacity of IMAC for stromal invasion [1, 9, 25]. However, we observed more intense cytoplasmic ANX A2 expression in the invasive component in the present IMAC case, thus suggesting that this protein is involved in stromal invasion [9, 12, 13]. Therefore, we presume (1) that whether IMAC tumor cells detach into alveolar spaces or infiltrate toward the stroma depends on the amount of secreted ANX A2, (2) that the stromal invasion of IMAC requires a stronger cytoplasmic expression of ANX A2, and (3) that extracellular membrane-bound ANX A2 enhances the aggressiveness of stromal invasion in AC [14, 15, 25, 26].

We note that weaker ANX A2 positivity was observed at the cell–cell borders in the detachment site, even in our previously reported case [9]. The expression of ANX A2 at the cell–cell borders is possibly related to the adhesions between tumor cells and the suppression of tumor cell detachment [9, 12, 13]. In other words, little or no expression of ANX A2 at the cell–cell borders might facilitate tumor cell detachment. Thus, we presumed that the lepidic or papillary component of the present tumor harbors a high capacity for detachment [9, 12, 13]. Nevertheless, the small groups of detached tumor cells showed a micropapillary pattern without the original monolayer arrangement. It has been suggested that tumor cells forming a micropapillary pattern acquire resistance to anoikis (cell detachment-induced apoptosis) and facilitate anchorage-independent growth [27]. Therefore, the morphological change of detached tumor cells is considered to be a defense reaction against the loss of interaction with the BM [11, 27]. It has been known that ANX A2 is released extracellularly during various stresses, including hypoxia [13], which is consistent with the findings that tumor cell aggregates floating within the alveolar spaces do not show a distinct ANX A2-immunopositivity.

Some reports have suggested that neutrophils and tumor-associated macrophages and fibroblasts promote STAS/AM [28, 29]. In the present case, we did not notice a particular infiltration of any of those cell types into the tumor. Although we agree that the tumor microenvironment is an important factor in STAS/AM, the present case suggests that the tumor cells can induce STAS/AM directly.

In this case, we consider that ANX A2 was only weakly involved in mediating the adhesions between tumor cells and in the anchoring of floating tumor cells to the alveolar wall. Conversely, abundant MUC 5AC was detected within and outside the tumor cells. In an IMAC with gastric lineage, MUC 5AC is thought to play a major role in the formation of contacts with the alveolar wall [30]. We assume that upon attaching to the alveolar wall, the round-shaped tumor cell aggregates stretch to form a monolayer along the alveolar wall in a manner independent of ANX A2.

It remains unclear whether the right lung tumor originated as a second primary lesion or was caused by an AM of the left lung tumor [17, 31, 32]. A similarly rapid clinical course was also reported in a previous case of multifocal IMAC [32]. Given that we did not genetically confirm monoclonality between the tumors and could not detect a recurrence in the ipsilateral lung, the right tumor might instead have been another lesion of multifocal IMAC.

This report had some additional limitations. Particularly, we analyzed only two cases, including our previously reported case. Moreover, our analysis lacked a functional assay and an immunohistochemical investigation of various BM matrices. Further investigations are needed to confirm our suppositions.

In conclusion, our findings from this case suggest the following points. (1) ANX A2 plays a dual role in tumor progression and suppression according to its subcellular localization. (2) The subcellular localization of ANX A2 depends on the characteristics of the tumor cell, including the biomarker immunophenotype and tumor cell growth pattern. (3) In the gastric lineage, ANX A2 secretion induces tumor cell detachment by degrading the BM. (4) The expression of ANX A2 (or lack thereof) at the cell–cell borders influences the detachment site.
